# A systematic review and meta-analysis of the catastrophic costs incurred by tuberculosis patients

**DOI:** 10.1038/s41598-021-04345-x

**Published:** 2022-01-11

**Authors:** Ramy Mohamed Ghazy, Haider M. El Saeh, Shaimaa Abdulaziz, Esraa Abdellatif Hammouda, Amira Mohamed Elzorkany, Heba Khidr, Nardine Zarif, Ehab Elrewany, Samar Abd ElHafeez

**Affiliations:** 1 Tropical Health Department, High Institute of Public Health, Alexandria University, Alexandria, Egypt; 2 Community Medicine Department, Faculty of Medicine, University of Tripoli, Tripoli, Libya; 3 Ministry of Health and Population, Alexandria, Egypt; 4 Epidemiology Department, High Institute of Public Health, Alexandria University, Alexandria, Egypt

**Keywords:** Diseases, Health care

## Abstract

One of the strategies of the World Health Organization End Tuberculosis (TB) was to reduce the catastrophic costs incurred by TB-affected families to 0% by 2020.Catastrophic cost is defined by the total cost related to TB management exceeding 20% of the annual pre-TB household income. This study aimed to estimate the pooled proportion of TB affected households who incurred catastrophic costs. We searched PubMed, SciELO, Scopus, Embase, Google Scholar, ProQuest, SAGE, and Web of Science databases according to Preferred Reporting Items of the Systematic Reviews and Meta-Analysis (PRISMA) guidelines till November 20, 2020. Eligible studies were identified and data on catastrophic costs due to TB were extracted. We performed a meta-analysis to generate the pooled proportion of patients with TB facing catastrophic costs. From 5114 studies identified, 29 articles were included in the final analysis. The pooled proportion of patients faced catastrophic costs was (43%, 95% CI [34–51]). Meta-regression revealed that country, drug sensitivity, and Human immune-deficiency Virus (HIV) co-infection were the main predictors of such costs. Catastrophic costs incurred by drug sensitive, drug resistant, and HIV co-infection were 32%, 81%, and 81%, respectively. The catastrophic costs incurred were lower among active than passive case findings (12% vs. 30%). Half (50%) of TB-affected households faced catastrophic health expenditure at 10% cut-off point. The financial burden of patients seeking TB diagnosis and treatment continues to be a worldwide impediment. Therefore, the End TB approach should rely on socioeconomic support and cost-cutting initiatives.

**PROSPERO registration**: CRD42020221283.

## Introduction

Tuberculosis (TB) infection is one of the top ten causes of death, with more than one million deaths worldwide in 2019^1^. According to the 2020 World Health Organization (WHO) report, Africa region had the highest incidence of TB (220/10^5^), followed by the South-East Asian region (211/10^5^), the East Mediterranean region (112/10^5^), and Western Pacific region (93/10^5^)^2^. At a country-based level, the number of reported new cases was the highest in India (26%), followed by Indonesia (8.5%), China (8.4%), Philippines (6.0%), Pakistan (5.7%), Nigeria (4.4%), Bangladesh and South Africa (3.6% for each)^3^.

On September 26, 2018, the WHO’s End TB Strategy was set to reduce TB incidence and deaths by 90% and 95%, respectively, and to find TB missing cases by active case finding (ACF) instead of passive case finding (PCF). The ACF refers to systematic identification and screening of people with presumptive TB among high-risk groups, using rapidly used screening tools or tests. On contrary, PCF entails visiting health services for diagnosis^4,5^.

The WHO also recommended that all patients with TB or their families should not be impeded by catastrophic costs incurred due to TB to complete their treatment^6^. Catastrophic costs are the total direct and indirect costs that reach or exceed 20% of the pre-TB-patient or household’s annual income^6^. Direct costs represent either the medical cost (consultation fees, diagnostic tests, and treatment) or nonmedical cost (transportation, accommodation, and increased food needs). Indirect costs include lost wages due to unemployment, time spent away from work, and associated loss of productivity. Moreover, patients incur huge costs in the pre-treatment phase to cover consultations and laboratory tests, symptomatic treatment, antibiotics trial, and hospitalization^7^. An important segment of the financial hardship is dissaving, which means reduced financial strength of a household, or engaging the household in damaging financial coping strategies. This reduces the financial capacity and their ability to cope with the financial shocks and casts them into the poverty trap^8^. Dissaving can take many forms, such as availing a loan, taking children out of education, selling assets, and reducing consumption to below basic needs to cope with health-related expenditure^7–9^.

Such expenses can impede their access and adherence to treatment, affect health outcomes, increase the risk of disease transmission, and add to the household`s economic burden. These added expenses were exaggerated by the coronavirus disease (COVID-19) pandemic^10^. Patients with TB incur expenses that, on average, equal half of their yearly income in some low- and middle-income countries. Moreover, TB disproportionately affects the lowest section of society. The poverty-aggravating consequences of TB are, thus, most severe for those who are already vulnerable^11^. Catastrophic costs are affected by several factors, such as patient age and sex, socioeconomic status, Human immuno-deficiency virus (HIV) co-infection, and being infected with multidrug-resistant TB (MDR-TB) that does not respond to at least isoniazid and rifampicin^11,12^.

The WHO has developed a cost survey of patients with TB to properly assess the total costs and proportion of patients facing catastrophic costs. This tool provides a standardized methodology for cross-sectional surveys in TB affected countries^13^. Many studies have used this cost survey to report catastrophic costs, catastrophic health expenditure, or hardship in financing faced by patients with TB^14–16^. Some studies calculated the catastrophic costs incurred for drug sensitive, MDR, or HIV co-infection^16–18^. Other studies have estimated these costs by adopting different case finding strategies (ACF versus PCF)^19,20^. In response to the reported catastrophic costs, the Global TB Program endorses social protection initiatives as cash transfers, food assistance, disability grants, and health insurance. These initiatives were run in parallel to the Universal health coverage initiatives^11,21,22^. Data on the pooled prevalence of TB patients suffering from catastrophic costs has not been aggregated through meta-analysis. We therefore conducted this systematic review to estimate the pooled proportion of patients with TB who incured catastrophic costs and identify the predictors of these costs among patients and their households.

## Method

We performed this systematic review and meta-analysis according to the Preferred Reporting Items of the Systematic Reviews and Meta-Analyses (PRISMA) guidelines^23^. Our research protocol was registered in PROSPERO (registration no. CRD42020221283).

### Data source and search strategy

We searched EMBASE, Scopus, EBSCO, MEDLINE central/PubMed, ProQuest, SciELO, SAGE, Web of Science, and Google Scholar databases for articles without timeframe, geographical or language restrictions till November 20, 2020 by two authors (ShA & NZ). RMG and SA re-ran the data-base search to check the search strategy and number of citations reported. In addition, they checked the number of citations exported to the reference manager. Highly focused and sensitive search strategies were developed by RMG after approval of PubMed Help Disk. The search terms include “tuberculosis “OR “Mycobacterium tuberculosis” OR “Koch’s disease” AND “catastrophic cost”. (Supplementary Table 1). We searched reference lists from included publications by hand and contacted researchers who are expertise in these surveys to assist in identifying any relevant publications.

### Study selection and eligibility criteria

We included observational studies that reported the proportion of patients suffering from catastrophic costs during the intensive (first 2 or 8 months of treatment in drug sensitivity (DS) or MDR, respectively), or the continuation phases of TB treatment.

Four authors (AME, ShA, NZ, and EE) independently screened titles and abstracts for relevant studies. We excluded non-observational studies, case reports, editorial, reviews, letters, and studies that did not report income because the catastrophic costs could not be calculated or when catastrophic costs were not calculated at the individual level (when the total direct and indirect costs incurred by all patients divided by the total income).

Two authors (AME, HE) independently assessed the retrieved abstracts and the full texts of these studies to determine eligibility according to the inclusion criteria. Disagreements were resolved through discussion and consensus, or through consultation with a third reviewer (SA), who solved these differences based on study judgements.

### Data extraction and analysis

Three authors (RMG, AME, HE) extracted the following data from eligible studies: country, study design, population criteria (age, sex, drug sensitive/resistant), treating facility (public/private sector) strategy of case finding (ACF/PCF), tool used to identify the catastrophic costs, and the catastrophic total costs and its determinants at different cut-off points.

### The outcomes and definitions

The primary outcome was the proportion of patients with TB and their households who incurred catastrophic costs. It was defined as the total direct and indirect costs because of TB reaching or exceeding 20% of the patient’s or household’s pre-treatment annual income^6^. Additionally, we addressed the main predictors of catastrophic costs and different coping strategies. Finally, we assessed the catastrophic costs among patients according to their drug sensitivity as DS or MDR (with or without HIV) and strategy of case finding (ACF versus PCF).

Secondary outcomes were the proportion of the direct costs to the total costs of TB treatment among DS or MDR, with or without HIV, catastrophic health expenditure (CHE; defined as the direct costs that reach or exceed 40% of patient’s capacity to pay or 10% of their household income)^24^, and the different coping strategies.

#### Study quality assessment

The Newcastle–Ottawa Scale was used to classify the quality of studies as very good studies (9–10 points), good studies (7–8 points), satisfactory studies (5–6 points), and unsatisfactory studies (0–4 points)^25^.

#### Publication bias

We assessed publication bias by visual inspection of funnel plots and Egger’s regression test.

#### Statistical analysis

Owing to the heterogeneity between studies, the proportion of catastrophic costs among patients with TB was pooled using the random effects model. Owing to the heterogeneity between studies, the proportion of catastrophic costs among patients with TB was pooled using the random effects model^26^.

#### Assessment of heterogeneity

Heterogeneity was assessed using the chi-square test on n-1 degrees of freedom, with an alpha of 0.05 considered for statistical significance and Cochrane-I-squared (I^2^) statistic. I^2^ values were classified as follows: 0 to 40%, might not be important; 30% to 60%, may represent moderate heterogeneity; 50 to 90%, may represent substantial heterogeneity; 75% to 100%, considerable heterogeneity^27^. Sources of heterogeneity, for identifying the possible effect modifiers on the pooled analyses, were explored using the following techniques: 

- **Find-out outliers:** If the study's confidence interval does not coincide with that of the pooled effect, it is considered an outlier. The size of the outlier has a substantial effect, and it deviates considerably from the overall effect. High-sampling-error studies vary significantly from the pooled result. However, because the confidence intervals of such studies are wide, there is a greater chance that the confidence intervals may overlap with one of the pooled effects. This basic outlier elimination technique is implemented using the find outliers function (dmetar) package. It seeks outlying studies in a (meta) item, eliminates them, and then recalculates the result (Supplementary Figure 1).
- **Sensitivity analysis:** We used the metafor R tool to conduct a leave-one-out sensitivity analysis. In this method, we recalculate the meta-analysis results K times; each time excluding one study. The influence () function includes a set of leave-one-out diagnostic tests that help identify of influential studies. This analysis also includes a categorization of what is regarded as influential. We used I^2^ to sort the studies in the plot. We identified studies with the highest heterogeneity and the final heterogeneity after excluding these studies (Supplementary Figure 2). We also created a Baujat plot, which compares the total heterogeneity contribution of each study to its effect on the aggregated outcome^26,28^ (Supplementary Figure 3).
- **Graphic Display of Heterogeneity (GOSH) plots**^29^: we fit the same meta-analysis model to all possible subsets of our included studies. In contrast to the leave-one-out method, we did not only fit K models, but also modelled for all 2* k* − 1possible study combinations (Supplementary Figure 4).

#### Subgroup analysis

We categorized the catastrophic costs at 20% for ACF and PCF patients, according to the country where the studies were conducted (inside/outside) India.

#### Meta-regression

We studied the impact of the country where the survey was conducted (high versus low incidence of TB)^30^, quality of the study, sex, and population criteria (DS, drug resistant with or without HIV) on the size effect of studies to explain the substantial heterogeneity.

## Results

### Search results

**Figure 1:**
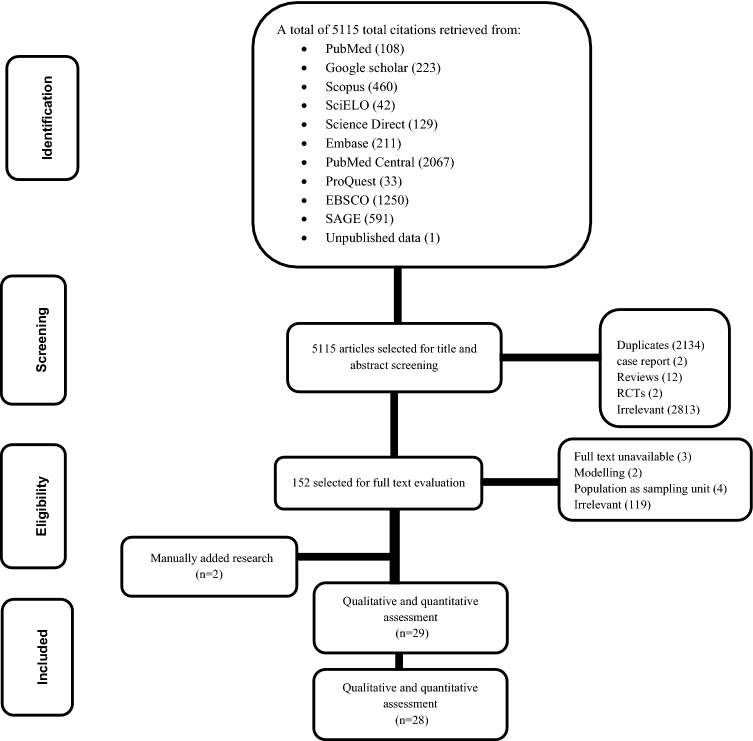
PRISMA flow-charts of studies included in meta-analysis of catastrophic costs among patients with tuberculosis.

Figure 1 showed the flow diagram of the selection process. The database search yielded a total of 5114 potentially relevant articles. After title and abstract screening, we excluded 2134 duplicates (1922 by Endnote, 212 manually), 2813 irrelevant articles, 12 reviews, 2 randomized control trials, and 2 case reports. Overall, 152 articles were eligible for full text screening. Two additional citations were found through manual search. Qualitative analyses included 29 articles; one study was omitted owing to its unsatisfactory quality. Finally, 28 studies were included in quantitative analysis. The inter-rater agreements for title and abstract screening, inclusion, quality assessment were κ = 0.8, 0.95, and 0.8, respectively.

### Study characteristics

Among the 29 studies included in the qualitative and quantitative analyese; six studies were from India, five were from China, four were from Indonesia, two were from Uganda and South Africa, and one was from Egypt, Zimbabwe, Nepal, Lao PDR, Ghana, Pakistan, Vietnam, Cambodia, Peru, and Cavite. Most studies were conducted at public health sectors, except for the study by Prasanna, et al.^31^, and four studies were conducted at both private and public sectors^14,19,32,33^. Only one study did not specify whether patients were treated under private or governmental sector^34^. The tools used for estimation of the cost survey were either WHO-TB cost survey tool^6,17,32,33,34–43^, the adapted WHO tool to Indonesian context^14,34^, structured questionnaire^19,44–46^, pre-coded interview scheduled^20^, tool of stop TB partnership^15,31,47^, headcount tool^48^, Lumley T. survey^16^, or TB coalition tool^18^. However, two studies did not mention the tool used^49,50^.

### Quality assessment

Paper quality was very good in one study^38^ and good in thirteen studies^6,18,19,31–34,36,37,41,42,44,45^. Fourteen studies were of satisfactory quality^14–17,20,35,39,40,43,45–48,50^ and one was unsatisfactory^49^ (Table 1).

**Table 1:** Studies that addressed catastrophic costs included in systematic review analysis.

Author, Year/Country	Study design	Population criteria study duration	Study setting	Sample size/Sex/Age	Tool used in cost estimation	Catastrophic costs (cut-off point) Predictors of CC	Quality interpretation
Shewade, 2018/ India^19^	Community-based cohort study	Sputum + ve pulmonary TB ACF&PCF 3/2016 – 2/2017	Both public and private sectors	N = 465 Male: 66% Age (years): 42 ± 17	Structured questionnaire	ACF:10.3% PCF: 11.5% (20%) Predictors: not mentioned	8 (Good)
Muniyandi, 2020/ India^33^	Community based cross-sectional study	Pulmonary and extrapulmonary TB patients registered in NTCP 2/2017 -3/2018	Both public and private sectors	N = 384 Male:67% Mean age 38.4 ± 16	WHO-TB cost survey	31% (20%) Predictors: Lower socioeconomic segments	7 (Good)
Wingfield, 2016/ Peru^44^	Community-based Prospective cohort study	Any patient treated with the Peruvian NTCP DS & MDR 2/2014 – 8/2014	Public sector	N = 876 Male: 59% Age ≥ 15 years	Questionnaire	39% (20%) Predictors: Inadequate nutrition, severe TB, hidden costs and adherence	7 (Good)
Muniyandi, 2019/ India^20^	Community-based cross-sectional study	ACF vs PCF 10/2016—3/2018	Public sector	N = 336 Male :77% Age ≥ 15 years	Pre-coded interview schedule	PCF:29% ACF:9% (20%) Predictors: not mentioned	5 (Satisfactory)
Fuady, 2020/ Indonesia^14^	Hospital-based cohort study	Treatment duration ≥ 1 month or completed treatment since < 1 month DS only 7–9/ 2016	Both public and private sectors	N = 252 Male: 54% Age ≥ 18 years	Tool adapted according to the Indonesian context	46% (10%) 38% (15%) 33% (20%) 26% (25%) 22% (30%) 17% (35%) Predictors: treatment duration, and additional visits	5 (Satisfactory)
Mullerpattan, 2018/ India^49^	Hospital-based cross-sectional study	Drug resistant-TB, hospitalized patients 8/2015 – 2/2016	Private sector	N = 50 Male: 30% Mean age = 30 years	Not mentioned	68% (20%) 78% (10%) Predictors: not mentioned	3 (Unsatisfactory)
Lu, 2020/ China^45^	Both community and hospital- based cross-sectional study	Culture-confirmed DS pulmonary TB 12/2014 – 12/2015	Public sector	N = 248 Male:54.9% Mean Age = 34 (IQR 26–49)	Standardized questionnaire	22.2% (20%) Predictors: not mentioned	6 (Satisfactory)
Prasanna, 2018/ India^31^	Both community and hospital-based Mixed methods	Both newly diagnosed and previously treated TB patients registered for treatment under NTCP DS 1/12/2016—31/1/2017	Private sector	N = 102 Male: 69% All ages	Estimate TB, Patient’s Costs developed by the Poverty SWC of the Stops Partnership	49% (10%) 32% (20%) Predictors: Age, HIV status and Hospitalization	8 (Good)
Fuady, 2018/ Indonesia^34^	Primary health care centers linked to NTCP cross-sectional survey	Patients treated 1 month or finished treatment since < 1 month Not Extra-pulmonary TB TB vs MDR-TB (poor vs non poor) 7–9/2016	Not mentioned	N = 346 (282 TB—64 MDR) Male: 55% Age: ≥ 18 years	Adapted Bahasa Indonesia version	DS 36% [Poor 43%, Non poor 25%] MDR-TB 83% (20%) Predictors: Travel costs, food / nutritional supplementation costs and income loss	8 (Good)
Yang, 2020/ China^36^	Both community and hospital- based cross sectional study	Pulmonary TB confirmed by sputum culture Rifampicin sensitive, MDR 9–10/2018	Public sector	N = 672 Male:64.3% Median age = 41 years	WHO-TB cost survey	46% (15%) 37.1% (20%) 30.2% (25%) Predictors: Age, Senior school or above, minimum living security household, employment status, household economic status, patient delay, medical care outside the city, hospitalization, MDR	8 (Good)
Chittamany,2020/Lao PDR^37^	Hospital-based Cross-sectional study	TB patients on treatment in intensive (> 14 days) or continuation phase People treated under NTCP, Pulmonary and extra-pulmonary, HIV, MDR-TB 12/2018- 1/2019 & 5–6/2019	Public sector	N = 848 Male:59.7% Mean age = 50.4 years	WHO-TB cost survey	Total 62.6% DS-TB 62.2%, DR-TB 86.7%, TB -HIV Co-inf. 81.1%, at (20%) Predictors: Food & nutritional supplements, income loss, treatment phase and educational status	8 (Good)
Viney, 2019/ Indonesia^38^	Hospital- based cross- sectional study	Any patient received treatment ≥ 2 weeks 10/2016 – 3/2017	Public sector	N = 457 Male: 50.6% Age = 32 years (IQR 22–52)	WHO-TB cost surveys	83% (20%) Predictors: Income loss & nutritional supplements, travel and medical costs after diagnosis	9 (Very good)
Wang, 2020/ China^48^	Hospital-based cross-sectional study	TB-MDR finished 1 year of treatment MDR-TB 1–8/ 2018	Public sector	N = 161 Male:68.9% Age = 36 years (IQR 26–48)	Headcount tool	87% (20%) Predictors: Low household income, absence of students in a family, LOS, male gender, job and productivity loss	5 (Satisfactory)
Muttamba, 2020/ Uganda^35^	Hospital-based cross-sectional study	Started treatment ≥ 2 weeks DS & MDR-TB 2017	Public sector	N = 1178 Male:62.7% All ages	WHO-TB cost surveys	53% (20%) Predictors: Transport, symptom relieving medications, food and loss of income	5 (Satisfactory)
Pedrazzoli, 2018/ Ghana^39^	Hospital-based cross- sectional study	Patients started treatment ≥ 2weaks DS & DR-TB, HIV 2016	Public sector	N = 691 Male:67.3% Median age = 41 years (IQR 29–52)	WHO-TB cost surveys	64.1% (20%) Predictors: Income loss, DR-TB & nutritional supplements	5 (Satisfactory)
Xu, 2019/China^46^	Hospital-based cross-sectional study	DS, pulmonary TB, under NTCP 3–6/ 2017	Public sector	N = 1147 Male:70.7% Median age = 51 years (IQR 12- 89)	Structured questionnaire	11.7% (20%) Predictors: Region, residence and insurance	6 (Satisfactory)
Ikram, 2020/ Pakistan^40^	Hospital-based cross-sectional study	TB- patients diagnosed > 3 months Pulmonary & DS, without HIV, hepatitis, nor DM	Public sector	N = 400 Male:47% Median age = 30 years (IQR 22–49 .50)	WHO-TB cost surveys	67% (20%) Predictors: Availability of paid sick leave, number of follow up visits and job loss	5 (Satisfactory)
Nhung, 2018/ Viet Nam^32^	Community-based cross-sectional study	(DS-TB & MDR-TB) including children Started treatment at least 2 weeks All ages DS & MDR-TB 7–10/2016	Both public and private sectors	N = 735 Male:75.9% Median age = 47 years (IQR 35–58)	WHO-TB cost surveys	Total 63%, 48%, 35% MDR 98%, 98%, 39%, DS 59.6%, 43% 30% COP:(20%), (30%), (40%) Predictors: Purchase special foods, travel, nutritional supplements, and accommodation	7 (Good)
Morishita, 2016/ Cambodia^50^	Both hospital and community-based cross-sectional comparative study	New pulmonary TB patients without unfavorable treatment outcomes & retreatment ACF vs PCF 2012 -2013	Public sector	N = 208 (108 ACF + 100 PCF) Male: 51.9% ACF: 48.1% PCF: 56% Median age: ACF = 55 (IQR 43.8–68) PCF = 52.5 (IQR 45–62.3)	–	ACF 54.6% 36.1% 24.1% 17.6% PCF 63% 45% 34% 21% COP: (10%) (20%) (30%) (40%) Predictors: Time spent for travel, waiting, consultation and hospitalization	6 (Satisfactory)
McAllister, 2020/ Indonesia^41^	Hospital-based cross-sectional study	Newly diagnosed pulmonary TB patients 10/2017 – 1/2019	Both public and private sectors	N = 69 Male:49.25% Age: ≥ 18 years	WHO-TB cost surveys	38.6% (10%) 26.5% (20%) 21.7% (25%), Predictors: not mentioned	7 (Good)
Tomeny, 2020/Cavite^17^	Hospital-based cross-sectional study	DS-TB vs MDR-TB 5–8/2016	Both public and private sectors	N = 194 Male:66% Age: ≥ 16 years	WHO-TB cost surveys	DS-TB 28% (20%) MDR-TB 80% (20%), Predictors: Travel, accommodation, and nutritional supplement	6 (Satisfactory)
Stracker, 2019/ South Africa^6^	Hospital-based cross-sectional study	2 months after diagnosis, transferred patients from other health care facilities to study clinics for treatment 10/ 2017–1/2018	Public sector	N = 237 Male:54% Age: ≥ 18 years	WHO-TB cost surveys	28% (20%) Predictors: Transport, treatment, income loss and time lost in seeking care	8 (Good)
Ruan, 2016/China^16^	Hospital-based cross-sectional study	MDR-TB 6–8/2012	Public sector	N = 73 Male: 48% All ages	Lumley T. Survey	78% (20%) Predictors: tests, nutrition, transportation, accommodation and time loss	6 (Satisfactory)
Mudzengi, 2017/ South Africa^18^	Hospital-based cross-sectional study	Diagnosed 3–5 month prior to the interview TB, HIV, or Both 4–10/ 2013	Public sector	N = 454 Male:36% Age: ≥ 18 years	TB Coalition tool	Total 60% (10%) TB/HIV 79% 67% 65% 64% 61% TB only: 55% 53% 47% 47% 45% HIV only: 72% 60% 55% 52% 49% COP: (5%), (10%),(15%), (20%), (25%)	7 (Good)
Gurung, 2019/ Nepal^42^	Hospital-based cross-sectional study	New and relapsed patients with TB (ACF vs PCF) 4–10/2013	Public sector	N = 99 Male:71% Age: ≥ 18 years	WHO-TB cost surveys	Total 52% PCF 61% ACF 44% (20%) Predictors: gender, age, disease category (new, relapse), poverty line, dissaving, financial and social impact	7 (Good)
Walctt, 2020/ Uganda^15^	Hospital-based retrospective cohort study	Spoke Luganda or English, confirmed active pulmonary TB Newly diagnosed TB 7–9/2017	Public sector	N = 224 Male:60.2% age: ≥ 18 years	Adapted version of Tool to Estimate Patients' Cost (stop TB partnership)	41.8% (20%) Predictors: Hospitalization, experience of coping costs, low-income status, age, education, HIV, unemployment, and female gender	6 (Satisfactory)
Rupani, 2020/ India^51^	Cross-sectional study	Patients not previously treated DS pulmonary TB 1/2019	Public sector	N = 458 Male:70% Median age = 35 (IQR 23–50)	WHO-TB cost surveys	14% (10%) 7% (15%) 4% (20%) Predictors: not mentioned	7 (Good)
Timire, 2020/ Zimbabwe^43^	Hospital-based cross-sectional study	Patients with DS or MDR TB 23/7–31/-8 2018	Public sector	N = 900 Male:56% Mean age: 36.9 ± 14.7	WHO-TB cost surveys	80% (20%) Predictors: Gender, Age, TB type, treatment phase, treatment delay HIV status, breadwinner, income quintile, and location of health facility	5 (Satisfactory)
Gadallah, 2018/ Egypt^47^	Hospital-based. prospective cohort study	New TB patients attending TBMUs for starting their treatment 1–6/2019	Public sector	N = 257 Male:61.9% Mean age: 38.3 ± 14.8 years	WHO-TB cost surveys	22.6% (10%) 24.1% (20%) 6.6% (30%) Predictors: Age, gender, unemployment, crowding index, governorates, income,	5 (Satisfactory)

*ACF* Active case finding, *PCF* Passive case finding, *SP* Smear Positive, *TB* Tuberculosis, *DS* Drug sensitive, *HIV* Human immunodeficiency virus, *LOS* Length of stay, *MDR* Multi-Drug Resistant, *NTCP* National TB Control Program, *SWC* Sub-Working Group, *TBMU* Tuberculosis medical unit.

### Publication bias

**Figure 2:**
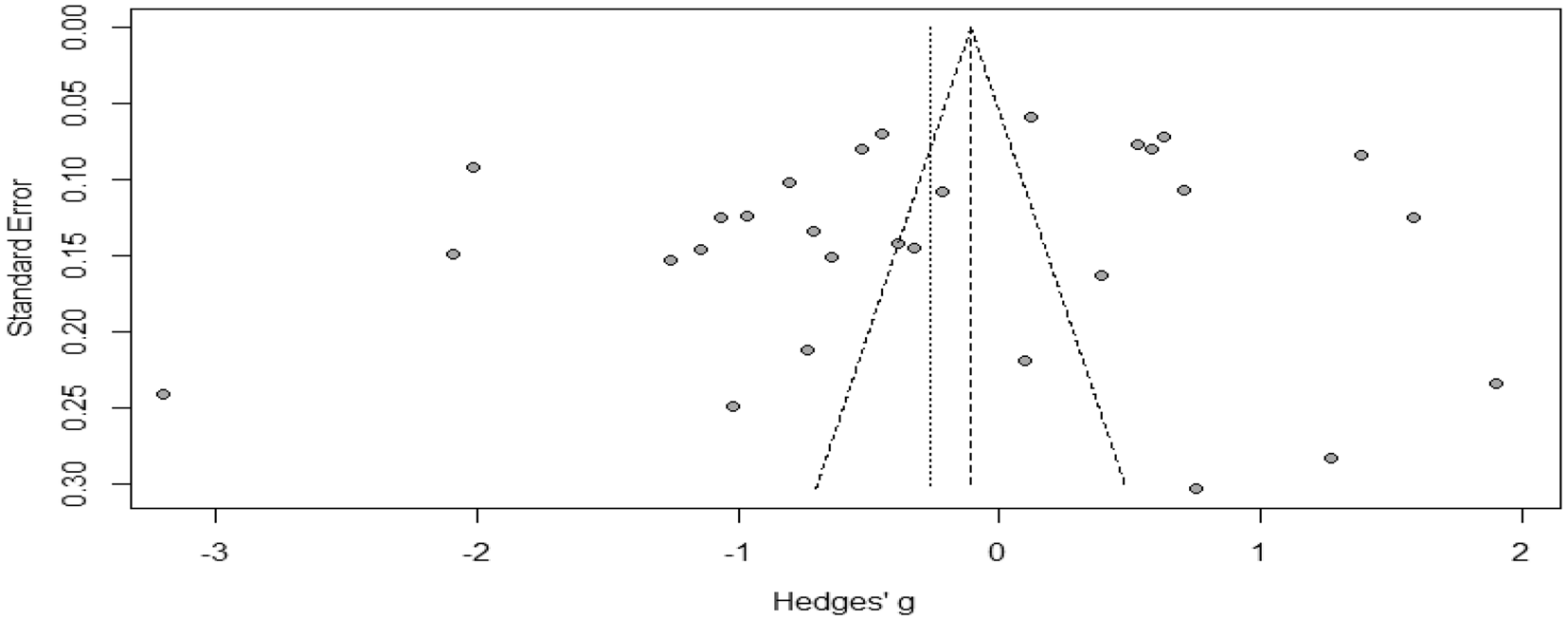
Funnel plot of studies included in the estimation of the proportion of tuberculosis patients and their households who faced catastrophic costs at a cut-off point of 20%.

Figure 2 showed that there was no publication bias as the funnel plot was symmetric. In addition, Eggers’ test was not significant [t = − 1.188, p = 0.24].

## Primary outcome

### Catastrophic costs at cut-off point 20%

**Figure 3:**
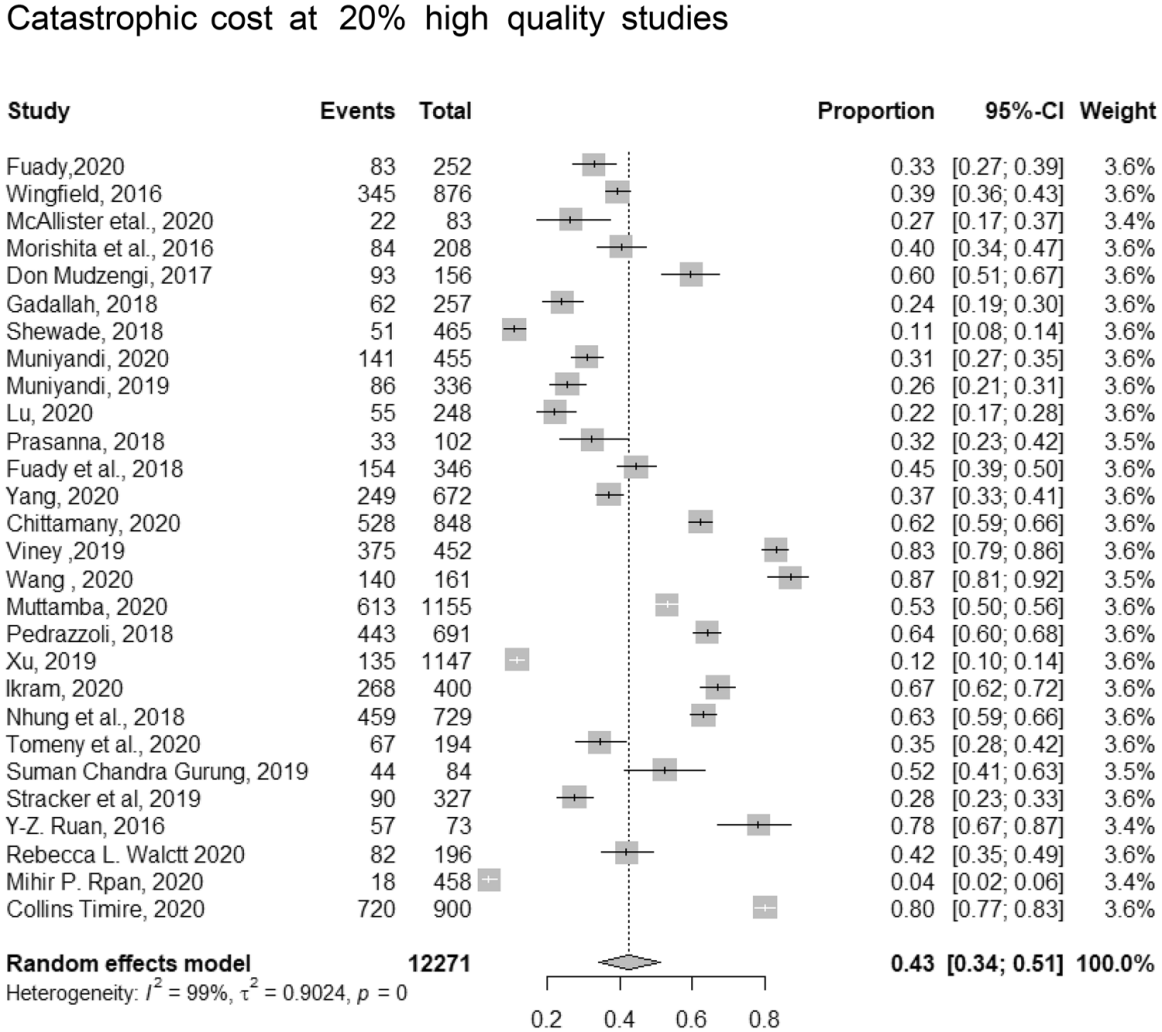
Pooled proportion of catastrophic costs incurred by TB patient and their household at a cut-off point of 20%.

The pooled proportion of catastrophic costs among 11,750 TB patients included in 28 studies at cut-off point of 20% was (43%, 95% CI [34–51]). The in between-study heterogeneity variance was estimated at ^τ2 = 0.90, *p* < 0.01 with an I^2^ value of = 99% (Fig. 3). We conducted meta-regression to identify the cause of this substantial heterogeneity. The predictors were sex, country where the study was conducted (had high incidence vs. none)^30^, DS (DS or MDR ± HIV), and quality of the study. The model was significant (*p* < 0.0127, R^2^ = 51.57%). This model explained more than 50% of the reported heterogeneity. The identified significant predictors were country (high vs low incidence) (β = − 0.194, p = 0.04) and type of patients regarding drug sensitivity (DS or MDR) and HIV co-infection (β = 0.289, p = 0.026).

### Predictors of catastrophic costs

The main predictors of catastrophic costs were food and nutritional supplements^37–39^, travel and transportation^35,36,50^, age category^31,36,44^, employment status^34,36,40,44,48^, the socioeconomic status^15,34,36,44,47,48,52^, MDR or HIV positive^31,36,39,52^, male gender^44,47,48^, and duration of hospitalization^15,31,36,48,50^.

### Coping strategy

To balance the enormous financial burden the families encountered by TB, they may adopt some coping strategies as borrowing money, availing loans, pledging gold and jewels, bringing their children out of schools, or selling assets^32,50^. All these approaches referred to as “dissaving” which is at the core of the hardship financing dilemma.

### Pooled proportion of catastrophic costs at cut-off point of 20% among different TB affected patients

#### Pooled proportion of catastrophic costs at cut-off point of 20% among TB drug sensitive

The pooled proportion of patients facing catastrophic costs was (39%, 95% CI [28–51]), and the reported heterogeneity was 99%. After removing the outliers^32,36–40,43,45,46^, the pooled proportion of 11 studies that recruited 3492 patients dropped to (32%, 95% [CI 29–35]). The pooled proportion of DS-TB patients facing catastrophic costs ranged from (24%, 95% CI [19–30]) in the study by Gadallah et al.^47^ to (42%, 95% CI [35–49]) in the study by Walctt et al.^15^ The heterogeneity of the included studies was as follows: I^2^ = 70%, *p* < 0.01 (Table 2).

**Table 2:** Pooled proportion of catastrophic costs at 20% among drug sensitive.

Study	Event	Total	Proportion	95%CI	Weight
Fuady, 2020	83	252	0.33	[0.27–0.39]	9.30%
Wingfield, 2016	295	783	0.38	[0.34–0.41]	12.20%
McAllister, 2020	22	83	0.27	[0.17–0.37]	5.10%
Gadallah, 2018	62	257	0.24	[0.19–0.30]	8.80%
Muniyandi, 2020	141	455	0.31	[0.27–0.35	10.90%
Prasanna, 2018	33	102	0.32	[0.23–0.42]	6.20%
Fuady, 2018	101	282	0.36	[0.30–0.42]	9.80%
Yang, 2020	197	586	0.34	[0.30–0.38]	11.50%
Tomeny , 2020	47	169	0.28	[0.21–0.35]	7.70%
Stracker, 2019	90	327	0.28	[0.23–0.33]	9.80%
Walctt, 2020	82	196	0.42	[0.35–0.49]	8.80%
Random effect model	1153	3492	0.32	[0.29–0.35]	
I^2^ = 70%					

#### Pooled proportion according to TB drug resistant

With a heterogeneity of 92%, the pooled proportion of TB affected household of MDR patients facing catastrophic costs among 1879 patients was (78%, 95% CI [86–86%]). After excluding the outliers^32,36,53^, the pooled proportion of patients facing catastrophic costs among 524 patients with MDR reached (81%, 95% CI [76–86]), I^2^ = 46%. The highest proportion (90%) was reported by Collin et al.^45^, while the lowest proportion (70%) was reported by Yang et al.^36^ (Table 3).

**Table 3:** Pooled proportion of catastrophic costs at 20% among drug resistant.

Study	Event	Total	Proportion	95%CI	Weight
Fuady, 2018	53	64	0.83	[0.71–0.91]	11.60%
Yang, 2020	39	56	0.7	[0.56–0.81]	12.90%
Chittamany, 2020	26	30	0.87	[0.69–0.96]	6.60%
Wang, 2020	140	161	0.87	[0.81–0.92]	15.00%
Pedrazzoli, 2018	50	66	0.76	[0.64–0.85]	13.10%
Tomeny,2020	20	25	0.8	[0.59–0.93]	7.30%
Collins Timire, 2020	44	49	0.9	[0.78–0.97]	7.80%
Ruan, 2016	57	73	0.78	[0.67–0.87]	13.20%
**Random effect model**	**429**	**1375**	**0.81**	**[0.76–0.83]**	
**I**^**2**^** = 46%**					

#### Pooled proportion of TB-HIV co-infected patients facing catastrophic costs at cut-off point of 20%

The pooled proportion of the 796 TB patients with HIV facing catastrophic costs at cut-off point of 20% was (76%, 95% CI [65–85%]), with a heterogeneity of 88%. After conducting leave-one out sensitivity analysis, the study by Mudzengi et al.^18^ was removed. The heterogeneity dropped to 0% and the pooled proportion of patients facing catastrophic costs has increased to (81%, 95% CI [78–84]) (Table 4).

**Table 4:** Pooled proportion of catastrophic costs at 20% among TB and HIV infected patients.

Study	Event	Total	Proportion	95%CI	Weight
Chittamany, 2020	100	123	0.81	[0.73–0.88]	17.80%
Timire, 2020	450	557	0.81	[0.77–0.84]	82.20%
**Random effect model**	**550**	**680**	**0.81**	**[0.78–0.84]**	
**I**^**2**^** = 0%**					

#### Pooled proportion of TB facing catastrophic costs at cut-off point of 20% through ACF

The proportion of patients facing catastrophic costs among the 491 patients exposed to ACF ranged from (9%, 95% CI [7–15%]) to (62%, 95% CI [45–77%]). After subgroup analysis based on the country where the ACF was implemented (inside/outside India), the pooled proportion was (10%, 95% CI [7–14%]), I^2^ = 0%) inside India and (48%, 95% CI [25–72%]), I^2^ 86% outside India. The difference in proportion of patients with TB incurring catastrophic costs at 20% was significant across the studied groups (*p* < 0.001) (Table 5).

**Table 5:** Pooled proportion of catastrophic costs at 20% among during active case finding after sub-group analysis.

Study	Event	Total	Proportion	95%CI
**Inside India**				
Muniyandi, 2019	10	110	0.09	[0.5–0.16]
Shewade, 2018	24	234	0.10	[0.7–0.15]
**Fixed effect model**	**34**	**342**	**0.1**	**[0.07–0.14]**
**Heterogeneity I**^**2**^** = 0%**				
**Outside India**				
Morishita, 2016	39	108	0.36	[0.27–0.46]
Gurung, 2019	24	39	0.61	[0.45–0.77]
Fixed effect model	63	247	0.26	[0.25–0.72]
**I**^**2**^** = 86.3%**				
**In-between groups P < 0.0001**				

#### Pooled proportion of patients with TB facing catastrophic costs during PCF

The proportion of patients facing catastrophic costs among 638 patients during PCF ranged from (12%, 95% CI [8–17%]) to (45%, 95% CI [35–55%]). The pooled proportion was (30%, 95% CI [17–48%]), I^2^ = 94%. We further subdivided the studies according to the studied country (inside/outside) India. The pooled proportion of TB households facing catastrophic costs outside India was (45%, 95% CI [37–53%]), I^2^ = 0% while inside India (19%, 95% CI [7–41%]), I^2^ = 95% (Table 6). After subgrouping the included studies according to the country where PCF was used (inside India/outside India), the difference in proportion of TB patients facing catastrophic costs at 20% was not significant across the studied groups (*p* = 0.063).

**Table 6:** Pooled proportion of catastrophic costs at cut-off point of 20% among during passive case finding after sub-group analysis.

Study	Event	Total	Proportion	95%CI
Morishita, 2016	45	100	0.45	[0.35–0.55]
Gurung, 2019	20	45	0.44	[0.30–0.60]
**I**^**2**^** = 0%**				
Shewade, 2018	27	231	0.12	[0.0–0.17]
Muniyandi, 2019	76	262	0.29	[0.24–0.35]
**I**^**2**^** = 95.2**				
**In between groups P = 0.06**				

## Secondary outcomes

### Proportion of direct costs to the total costs

#### Direct to total costs among drug sensitive

The proportion of the mean direct costs to the mean total costs were addressed in six studies; the pooled proportion of direct to total costs at catastrophic costs of 20% were not calculated because of high heterogeneity. The proportion was variable; Tomeny et al.^17^ and Timire et al.^52^ reported that catastrophic costs were 41% and 43%, respectively. However, a higher proportion (52%) was reported by Chittamany et al.^37^ and Nhung et al.^32^. Two other extreme values were reported by Fuady et al.^34^ and Muttamba et al.^35^ (33% and 65%, respectively).

#### Direct to total costs among multidrug resistance

The proportion of the mean direct costs to the mean total costs at cut-off point of 20% was addressed in seven studies ranged from 26% in Chittamany et al.^37^ to 93% in Yang et al.^36^. Low proportions were observed in the studies of Fuady et al.^34^, Tomeny et al.^17^, and Timire et al.^52^ with proportion of 32%, 34% and 49% respectively, while a high proportion was reported by Muttamba et al. (66%)^35^, and by Nhung et al. (68%)^32^. The pooled proportion of mean direct to total costs was difficult to assess because of the substantial heterogeneity which was not explained even after a meta-regression analysis.

#### Pooled proportion of direct costs to total costs in the case of ACF

The pooled proportion of the mean direct costs to the mean total costs was addressed in four studies and was (25%, 95% CI [16–37%]), I^2^ = 83%. After conducting leave one out sensitivity analysis, the study of Gurung et al.^42^, was removed, the pooled proportion dropped to (29%, 95% C1 [20–41%]), I^2^ = 55%. (Table 7).

**Table 7:** Pooled proportion of direct to total costs at catastrophic costs of 20% among active case finding.

Study	Event	Total	Proportion	95%CI	Weight
Morishita, 2016	110.5	399	0.28	[0.23–0.32]	57.70%
Shewade, 2018	12	4.5	0.8	0.28–0.99]	4.90%
Muniyandi, 2019	18	69	0.26	[0.16–0.38]	37.40%
Random effect model	**140.5**	**427.5**	0.29	[0.20–0.41]	
**I**^**2**^** = 55%**					

#### Pooled proportion of direct costs to total costs in case of passive case finding (PCF)

The pooled proportion of the mean direct costs to the mean total costs was addressed in four studies^19,20,42,50^ and was (37%, 95% C1 [31–42%]), I^2^ = 0% (Table 8).

**Table 8:** Pooled proportion of direct to total costs at catastrophic costs of 20% among passive case finding.

Study	Event	Total	Proportion	95%CI	Weight
Morishiita, 2016	206	535	0.39	[0.34–0.43]	33.60%
Shewade, 2018	26.9	28.4	0.94	0.98–0.90]	4.20%
Muniyandi, 2019	79	227	0.35	[0.29–0.41]	30.10%
Gurung, 2019	131.74	325.3	0.45	[0.35–0.46]	32.10%
**Random effect model**	**443.64**	**1115.7**	**0.37**	**[0.31–0.42]**	
**I**^**2**^** = 0%**					

#### Proportion of direct costs to total costs in the case of HIV and TB co-infection

The proportion of the direct costs to the total costs were addressed in two studies. Mudzengi et al.^18^ showed that the proportion of mean direct costs to the mean total costs was 30% among HIV and TB co-infection patients, while a higher proportion (59%) was reported by Chittamany et al.^37^. We couldn’t pool these studies because of the unexplained heterogeneity.

#### Proportion of mean direct costs to total costs

The pooled proportion of the mean direct costs to the mean total costs was addressed in 13 studies, which ranged from 4 to 87% (Supplementary Table 2).

#### CHE at 10% & capacity to pay at 40%

There were six studies calculated the CHE 10% and the capacity to pay (CTP) 40%.

#### Pooled proportion of CHE at 10%

The pooled proportion of the CHE at 10% was addressed in three studies. The pooled proportion of TB patients who incurred CHE was (45%, 95% CI [35–56%]), I^2^ = 93%. After leave one out sensitivity analysis, Fuady et al.^41^ was excluded, and the heterogeneity decreased to reach I^2^ = 28% and the pooled proportion has increased to (50%, 95% CI [47–54%]) (Table 9).

**Table 9:** Pooled proportion of Catastrophic Health Expenditure at 10%.

Study	Event	Total	Proportion	95%CI	Weight
Lu, 2020	132	248	0.53	[0.47–0.60]	26.80%
Muttamba, 2020	567	1155	0.49	[0.46–0.52]	73.20%
**Random effect model**	**699**	**1403**	**0.5**	**[0.47–0.54]**	
**I**^**2**^** = 28%**					

#### Pooled proportion of capacity to pay (CTP) at 40%

Three studies measured the CHE in relation to CTP. The pooled proportion of TB patients who face CHE was (63%, 95% CI [40–80%]), I^2^ = 96%. After conducting the sensitivity analysis, the heterogeneity was found to be = 0%, while the pooled proportion increased to (70%, 95% CI [64–76%]) (Table 10).

**Table 10:** Catastrophic Health Expenditure (Capacity to Pay at 40%).

Study	Event	Total	Proportion	95%CI	Weight
Wang, 2020	110	161	0.68	[0.61–0.5]	71.30%
Ruan, 2016	54	73	0.74	[0.62–0.84]	28.70%
**Random effect model**	**164**	**234**	**0.7**	**[0.64–0.76]**	
**Heterogeneity I**^**2**^** = 0%**					

## Discussion

Our meta-analysis showed that the proportion of patients facing catastrophic costs at a cut-off point of 20% was 43%; (32%, 95% CI (29–35)) among DS and (80%, 95% CI [74–85%]) among MDR). Patients with TB co-infected with HIV faced the highest catastrophic costs (81%, 95% CI [78–84]). Catastrophic costs were variable according to the strategy of case finding; ACF = (12%, 95% CI [9–16%]) versus PCF (42%, 95% CI [35–50%]). Among drug sensitive and drug resistant TB, the proportion of direct costs to the total costs ranged from 33 to 65%^17,32,34,35,37,52^ and 26–93%^17,32,34–37,52^ respectively. ACF incurred lower catastrophic costs than PCF (29%, 95% CI [20–41%]) versus (37%, 95% C1 [34–40%]). The direct to the total costs among TB and HIV co-infected patients ranged from 30%^18^ to 59%^37^. The CHE was (50%, 95% CI [47–54%]), and (70%, 95% CI [64–76%]) at 10% of household’s yearly income and 40% of their CTP, respectively.

### Catastrophic costs

The costs incurred by TB on some patients may be catastrophic and minimal for others. This is based on the household annual income. In the current study, we included 28 studies that addressed catastrophic costs among patients with TB at different thresholds points (30%, 25%, 20%, 10%, and 5%). Despite the absence of robust evidence on the sensitivity of the cut-off point at 20% to reflect the catastrophic costs, regardless of whether patients are drug sensitive or resistant; Fuady et al.^14^ established 15% and 30% as more consistent cut-off points for treatment adherence and success, respectively. In the current study, the proportion of TB-household patients facing catastrophic costs was 39%, which was considered very high compared with the targeted sustained developmental goals in 2020 (0%), thus more efforts and activities should be directed to reduce these costs. Diagnosis and treatment are provided free in many of the included countries under the umbrella pool of NTP; however, the treatment related expenditure is still very high. Yadav et al.^54^ illustrated that even with free services for TB care, 21.3% of the patients were exposed to hardship financing, thus recommending more innovative ways to increase the supported coverage of TB treatment in the countries. The study also suggests the use of hardship financing as an index to measure the effectiveness of TB control program. It is crucial to decrease the burden of catastrophic costs among patients with TB, as it results in poorer treatment outcome. Patients suffering from catastrophic costs had 2–4 times higher odds of treatment failure than those who do not^14^. This could be explained by the reduced access to treating health facility and treatment completion. Regarding the coping costs, the majority of household’s resort to different coping strategies to deal with the increased out-of-pocket costs and to compensate for the consequences of income loss. Those coping strategies include selling a property or livestock, taking loans, pledging jewels, dropping their children out of school, and cutting down their consumption to below basic needs^11^. Despite pooling of these studies’ outcome yielded substantial heterogeneity, the current study found that 51.57% of heterogeneity was mainly because of two predictors; the first was that some studies estimated catastrophic costs of DS and patients with MDR with or without HIV together. This factor played a major role in the heterogeneity, as it was clear that the catastrophic costs were dramatically higher among patients with HIV. The second predictor was the classification of the country where the study was conducted^30^. Two-thirds of the new cases of TB are reported in eight countries of the world, with India being the highest, followed by Indonesia, China, the Philippines, Pakistan, Nigeria, Bangladesh and South Africa. Consequently, we sub-grouped the studies according to the country where they were conducted; countries with high versus low TB incidence. In meta-regression analysis, the country where the study was conducted was the second major determinant of the different size effect.

The reported high incidence of catastrophic costs in many countries raised the need for social protection interventions. The most common social protection intervention is the cash transfer or cash assistance, which has already been implemented in many countries across the world, either conditionally or unconditionally^55^. Thus, it is supposed that the household can get better access to treatment and food. Other social protection interventions include disability grants, food baskets (food assistance), food or travel vouchers and social insurance^11^. Many countries have implemented reimbursement programs to help patients with TB to cope with the disease costs. However, these programs prioritize poorer and MDR^56^. The effect of this intervention is questionable. At a cut-off point of 20%, two studies have applied and calculated a catastrophic costs before and after reimbursement. Lue et al.^45^ reported a minimum change in the proportion of catastrophic costs; before reimbursement, the catastrophic costs were (22%) and declined to 19% after the reimbursement. In contrast, Fuady et al.^57^ showed a higher change in the proportion of catastrophic costs after the reimbursement. The intervention program effectively decreased catastrophic costs from 44 to 13%. Regarding cash transfer, Wingfield et al.^53^ reported that the proportion of TB households suffering from catastrophic costs was 30% and 42% among intervention and control groups, respectively. These findings indicate that this social support is not enough to mitigate the impact of TB. Consequently, households of TB patients should receive sufficient financial support that covers indirect costs (job lost) and direct costs (transportation, food, accommodation)^58^. Such social support should be proportionate to the income lost because of the high variability of the pre-treatment income. We speculate that developing newer treatment guidelines for TB of a shorter duration would be beneficial. At the bottom, providing free medication is insufficient to prevent the catastrophic costs. TB patients should receive transport vouchers, reimbursement schemes, and food assistance to reduce or compensate for such catastrophic costs. Furthermore, decentralization of patient supervision (including directly observed therapy), for example, through community-based or workplace-based treatment^59^, can reduce transport costs and income loss for patients^60^.

As expected, the catastrophic costs among MDR were higher than among DS, as DS patients receive treatment for shorter duration (6 months only), while MDR treatment extends to 24 months. Additional cost is incurred by MDR patients, such as the cost related to prolonged work absenteeism, need for daily injection, exposure to more side effects, and need for more investigation^61^.

### Direct costs to total costs

The mean total direct costs to the mean total costs were lower than the mean indirect costs among drug sensitive patients, HIV co-infected patients, while it was higher among drug resistant patients. This finding is essential to be considered when reimbursement strategies are implemented. Stakeholders should know which part of patient costs should be compensated. The direct costs dropped significantly if the ACF strategy was adopted instead of the PCF (29% to 37%) respectively.

### Determinant of catastrophic costs

Recognizing the determinants of catastrophic costs could provide an insight into approaches for mitigating catastrophic costs among the vulnerable TB patients and their households. The epidemiological consequence of TB is directly related to a country's socioeconomic profile. TB vulnerability is determined by biological variables (e.g., malnutrition, HIV infection, and age) and social factors (e.g., poor housing conditions, high population density, inhumane working conditions, and a lack of access to health services). Under many circumstances, numerous vulnerabilities occur simultaneously^62^. In this study, the main determinants of catastrophic costs were income loss as an impact of being diseased and food and nutritional supplements other than the patients’ regular diet habit^37–39^. In addition, travel and transportation affected the direct non-medical costs, thus increased the suffering of patients with TB^35^. Age also affected the proportion of patients with TB suffering from catastrophic costs, whether young^47^ or old^36^. Additionally, Kirubi et al.^63^ found that delayed treatment initiation was a major predictor of catastrophic costs. Approximately 24% of individuals with catastrophic expenses waited for more than four weeks following the onset of symptoms to start treatment. Severe symptoms, prolonged hospitalization, more expensive non-TB medication, or even more frequent visits to the facilities may explain why delayed treatment initiation was related. Health and social protection investments have minimized the negative health effects of TB. Moreira et al.^62^ emphasized the relevance of public social protection programs in mitigating the consequences of TB indicators in the pursuit of TB elimination.

### Catastrophic health expenditure

Out of the 28 studies, only six studies have been included with a clear measurement of the CHE (at cut-off point of 10% of their income and at cut-off point of 40% of their CTP). It was clear that many studies ignored CHE, despite its importance to understand the impact of these costs on treatment outcomes^45^. Two studies assessed the effect of reimbursements intervention on the CHE. Xiang et al.^64^ reported a 8% reduction in CHE, however, this reduction was not statistically significant. Similarly, Zhou et al.^65^ reported that the effect of reimbursement on CHE was minimal; only 12% reduction in CHE was achieved. To decrease the catastrophic expenditures national health financing systems must be designed and implemented, not only to allow people to access services when they are needed but also to protect households from financial catastrophe, by reducing out-of-pocket spending. Eventually, prepayment mechanisms should be developed, for instance, social health insurance, tax-based financing of health care, or some mix of prepayment mechanisms such as efficient reimbursement or cash intervention^66^.

### Strengths and limitations of the study

Our study has several strengths and limitations. The strengths include the wide sensitive search strategy and multiple studied outcomes. The limitations of this study was that different cut-off points were established by different studies to estimate the proportion of the households facing catastrophic costs using different tools. Second, a major challenge was that different studies estimated catastrophic costs due to TB, regardless of drug sensitivity (DS, MDR), co-infection with HIV, case finding strategy (ACF, and PCF). Third, all studies included subjects with confirmed TB. Costs for those ill patients with undiagnosed TB may add much to the already estimated values. Fourth, many of the included studies used the WHO cost survey tool which included patients only treated in the NTP, omitting patients treated in private sectors who represent a considerable proportion. Fifth, owing to the observational nature of the studies included, there was a risk of recall bias as well as a chance of reporting inaccurate significant relationships due to confounders. Sixth, the degree of heterogeneity across the studies was likewise substantial; so, the random effects method was used to generate the pooled data. In addition, we adopted several techniques to overcome it like meta-regression and subgroup analysis. Finally, the quality of a meta-analysis is determined by the quality of the included research; here, the quality of the majority of the included studies was graded as satisfactory or good.

## Conclusion

Regarding future global policy, our study provides an evidence for the high proportion of TB patients who are still facing catastrophic costs despite the free TB treatment policy. The proportion of patients facing catastrophic costs varies according to the type of TB, which is the lowest among DS, higher in MDR, and the highest among those with concomitant infection with HIV. Patients exposed to ACF incurred lower costs than those exposed to PCF. The direct costs (medical &non-medical) related to TB is not the only major contributor to the catastrophic costs, but indirect costs (Job and productivity lost) also represent a major contributor that should not be ignored. Overall, this study paves the way to effective cost mitigation in the context of the End TB Strategy. Effective management of the predictors of catastophic costs will eventually contribute to better community, clinical, and financial outcomes. It is clear that the global health system must make more efforts to achieve the zero catastrophic costs for TB by 2030. Future research should assess the effectiveness of reimbursement for TB patients on the reduction of the proportion of patients who face catastrophic costs. Furthermore, in an attempt to reduce the costs incurred by TB patients, researchers should develop more reliable diagnostic tools to reduce patients' need for frequent visits to healthcare facilities. They also have to study the impact of educational programs on TB patients' compliance with the prescribed medicine to lower the retreatment rates. Finally, NTP should monitor the financial and social status of patients treated and intervene as early as possible to protect them from incurring these catastrophic costs.

## Supplementary Information

Supplementary Information 1.

Supplementary Information 2.

## Data Availability

Data are available upon request by contacting the first author.
