# Supplementary material for: A systematic review and meta-analysis of the catastrophic costs incurred by tuberculosis patients
Source: Sci Rep. 2022 Jan 11;12:558. doi: 10.1038/s41598-021-04345-x (PMC8752613; doi:10.1038/s41598-021-04345-x)
Supplement: Supplementary file 1 — Supplementary Information 1. [file 41598_2021_4345_MOESM1_ESM.docx]

**Supplementary files**


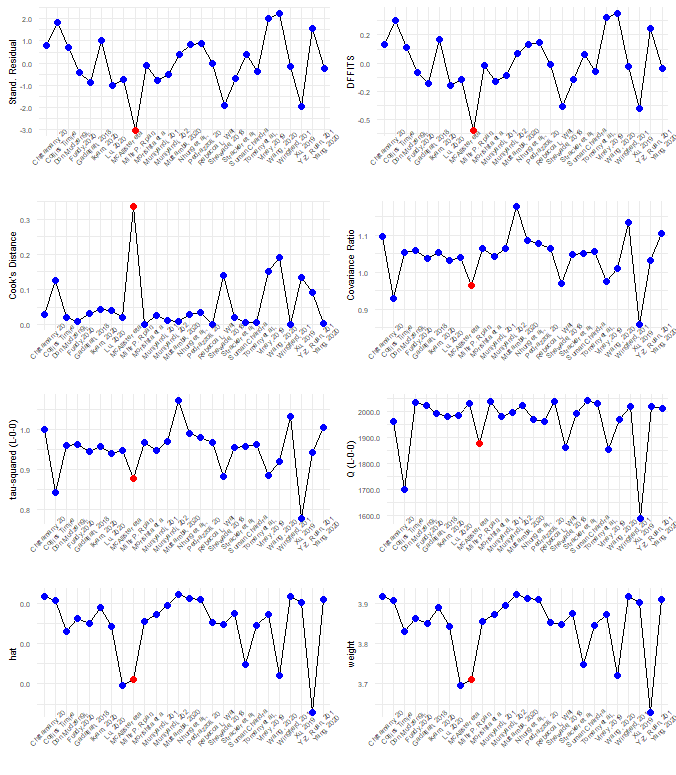
**Supplementary Figure 1**: Influence Diagnostics the primary outcome (catastrophic cost at 20%)


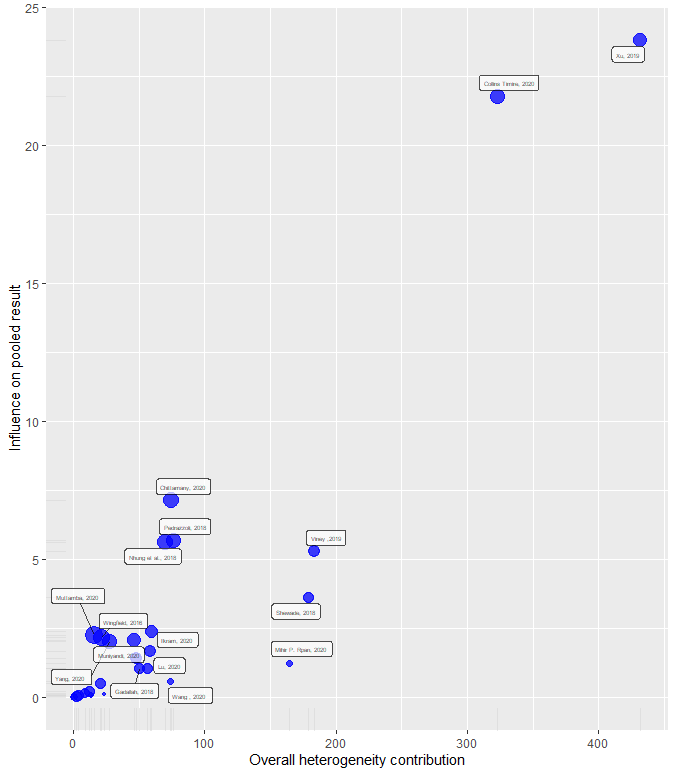


**Supplementary Figure 2:** Baujat plot of the primary outcome (catastrophic cost at 20%). Studies on the right side of the graph can be considered possibly important cases because they contribute significantly to the overall heterogeneity. Studies in the upper right corner of the plot may be especially influential because they have a great effect on both the estimated heterogeneity and the pooled effect.


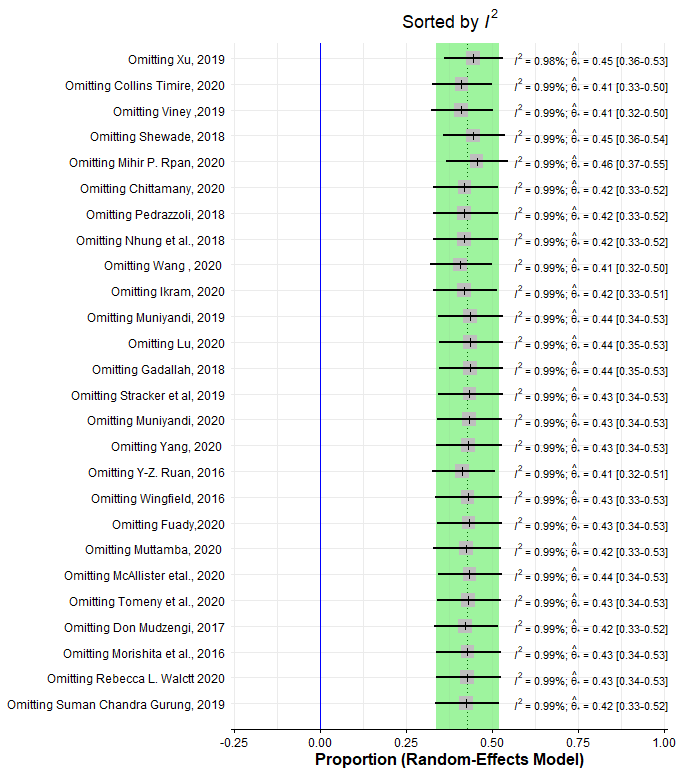


#### Supplementary 3: Leave-One-Out Meta-Analysis Results (ordered by heterogeneity low to high, as measured by I^2^) of the catastrophic cost at 20%


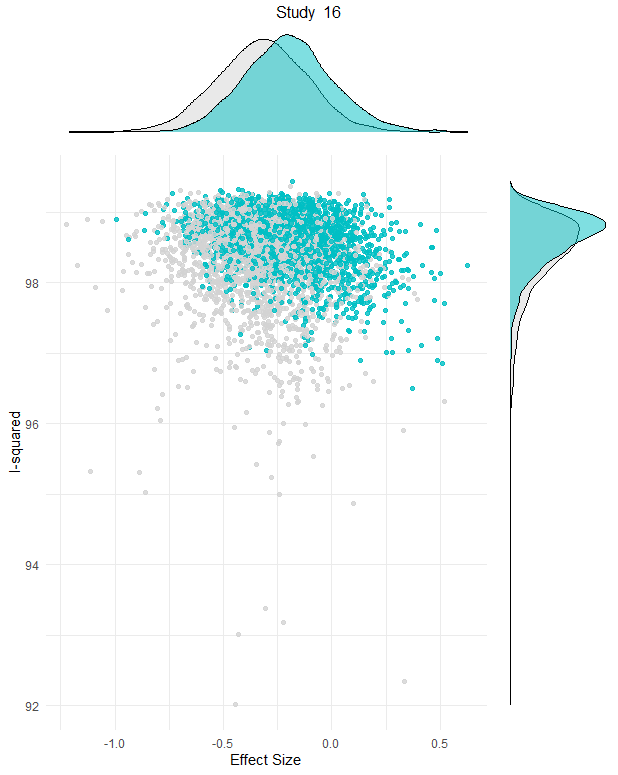


#### Supplementary Figure 4: GOSH analysis of studies that addressed the catastrophic cost incurred by TB at 20%. Most values are concentrated in a cluster with relatively high effects and high heterogeneity.
